# On the Medical as Well as Dietetic Properties of Common Salt

**Published:** 1818-05

**Authors:** John Marshall


*For the London Medical and Physical Journal.*

### *On the Medical as well as Dietetic Properties of Common Salt.* By JOHN MARSHALL, Esq.

THE following case may probably be considered as worthy insertion in your Journal, as it affords an interesting proof of the anthelmintic properties of *common salt.* The attention of the public has been lately attracted to the generally salubrious virtues of this substance by Sir Thomas Bernard, in his patriotic researches into the Salt Duties. In his work,* he has introduced an interesting letter from my friend, Dr. Paris, of Dover-street, which is more immediately connected with the object of this communication, and which I conceive to be of such medical importance as to deserve particular notice in a professional work; I must, therefore, beg to quote the passage.—"No idea can be formed of the distress of the lower orders of people in the inland parts of Cornwall, for *want of salt.* They are obliged to eat their potatoes without any relish, unless they can beg from the neighbouring farmer a little pickle: it is the universal clamour, '*if we could but have a little salt, we should be contented.*" They appear to have a desire for salt, almost as strong as the fatal passion for ardent spirits; and a degree of exhilaration and comfort is experienced after it, which it is difficult for those who have not witnessed it to conceive. Diseases of the *stomach* are the *prevalent* complaints of these poor people; and I am induced to believe, from observations made during an extensive practice, that children become *infested with worms,* by a diet of fish that is not properly salted."

On perusing Dr. Paris's account of this prevalent cause of disease and sickness, Sir Thomas Bernard observes, that "the mind naturally recurs to Lord Somerville's account (in his Address to the Board of Agriculture) of the effects of a dreadful punishment which formerly existed in a neighbouring country:—'In Holland, the ancient laws ordained men to be kept on bread alone, *unmixed with salt,* as the *severest* punishment that could be inflicted in their moist climate. The effect was HORRIBLE: these wretched criminals are said to have been DEVOURED BY WORMS, engendered in their own stomachs.'"

* See Case of the Salt Duties, with Proofs and Illustrations; by Sir Thomas Bernard, Bart.—London: John Murray, Albemarle-street. 8vo. December, 1817.

The following case, which occurred under my immediate notice and care, seems particularly worthy of attention on this occasion.

In the month of December last, I attended a lady who supposed herself attacked with symptoms of approaching apoplexy, or, to use her own words, "a fulness of blood—irregular circulation—with such a determination to the vessels of the head that she feared it would deprive her of life, or, at least, produce a paralytic seizure." With these impressions, being a little whimsical in her notions, and replete with nervous apprehensions, she frequently sent for a cupper, who, by her own desire, was directed to abstract from the back of her neck, at each operation, sixteen ounces of blood, and this was repeated five or six times in the course of twelve months. Previous to my seeing her, she had occasionally consulted Sir Walter Farquhar, who always endeavoured to dissuade her from having recourse to cupping, or any other sudden means of depletion. I was called to see her, in consequence of this impression being greatly increased by dizziness, attended with symptoms of indigestion, loss of appetite, a numbness of the right lower extremity, and a convulsive motion of the left eye-lids. She was strongly persuaded that cupping would be of the greatest service, to which I consented, and seven ounces only were obtained. The blood appeared the next day very thin, and its texture less firm than natural, which sufficiently marked the frequent application of the scarificator; I felt, therefore, much satisfaction at not having doubled the quantity. But I must now proceed to what I consider the most interesting part of the history of this case. After a week's attendance, she mentioned a symptom which she deemed of a most unpleasant nature, viz. worms, with which she had been troubled *all her life;* and these were *ascarides*, constantly voided in greater or less quantities, with the usual irritation in the rectum, which was at times intolerable. She had voided also *lumbrici*. Having just perused Dr. Paris's observations, in the work of Sir Thomas Bernard, I enquired whether she was in the habit of taking salt with her food; she replied, that she had an antipathy, from a child, to its use, &c. On entering minutely into a thorough investigation of her case, as regarded the total absence of salt, I found that even her cook was directed never to use salt on any account in her diet. About three years ago, she was attacked with a violent cough, that completely baffled the usual remedies, which were prescribed by a skilful hand on that occasion; nothing relieved the violent paroxysms of coughing but *eating:* after dinner (which was late) the cough entirely ceased for some time, and the more solid and copious the meal, the longer was the interval of quiet. One day, however, at noon, she coughed so violently that it caused her to vomit: the efforts of reaching were repeated three times, and each effort brought up several worms, which terrified her exceedingly, but from that moment the cough was relieved. I enquired, if she could recollect how long she had actually been troubled with worms; to which she emphatically replied, "she had been troubled with them as long as she could remember, and from her earliest infancy, and had never been free from them, although she was now sixty-six years of age." She had, in obedience to my wishes, eaten a little salt with her meals, but she could not undertake to go on with it, because it was so highly nauseous to her taste, and she hoped that I would devise some other means of subduing the worms: salted meats were suggested, such as ham, tongue, hung-beef scraped, &c.; and, it is curious to remark, that she had never eaten them in the course of her life, and she would rather endeavour to use salt with fresh meat than partake of them. Her complexion is pale; the teeth, considering her age, are remarkably good in front. This lady is generally habituated to exercise, and is a great walker: frequently walks, for amusement, a distance of fifteen or even twenty miles.

On mentioning this case, and Dr. Paris's observations, to a reverend divine, who resides at his deanery in Ireland, he favoured me with some additional remarks on this interesting subject. A singular fact, well known in Ireland among those who have the superintendance of horses, is, that salt is considered the most effectual and certain remedy for *bots* in that animal. He also informed me, that the poorer orders, subject to worms, used a dose of common salt as a popular remedy. In that country, where the duties on this article are not so excessive, the mode of fattening hogs is by sprinkling a little salt upon every meal, by which means they are found to fatten in half the period they would otherwise require. In some parts of that kingdom, the inhabitants are fully aware of the advantages in mixing it with manure.

I will conclude this communication, by reminding your readers, that Rush states, that he has administered many pounds of common salt as an anthelmintic remedy; the following is his formula:—℞. Sodæ Muriatis, ℥ij. Coccinellæ, ℈ij. M. Fiat Pulvis, detur drachma dimidia, pro dosi, tempore matutino.

I might extend this paper by some observations upon the antiseptic properties of salt. Dr. Paris, in his evidence before a committee of the House of Commons, upon the subject of the Salt Duties, has stated many interesting facts upon the subject; and, in your Journal for December 1809, you have recorded some cases from the pen of that scientific physician, in illustration of the antifebrifuge effects of muriatic acid.

12, *Half-Moon-street, Piccadilly;*
*April* 8, 1818.

